# Assessment of the Impact Resistance of a Composite Material with EN AW-7075 Matrix Reinforced with α-Al_2_O_3_ Particles Using a 7.62 × 39 mm Projectile

**DOI:** 10.3390/ma13030769

**Published:** 2020-02-07

**Authors:** Adam Kurzawa, Dariusz Pyka, Krzysztof Jamroziak, Marcin Bajkowski, Miroslaw Bocian, Mariusz Magier, Jan Koch

**Affiliations:** 1 Department of Lightweight Elements Engineering, Foundry and Automation, Faculty of Mechanical Engineering, Wroclaw University of Science and Technology, Smoluchowskiego 25, 50-370 Wroclaw, Poland; adam.kurzawa@pwr.edu.pl; 2 Department of Mechanics, Materials and Biomedical Engineering, Faculty of Mechanical Engineering, Wroclaw University of Science and Technology, Smoluchowskiego 25, 50-370 Wroclaw, Poland; dariusz.pyka@pwr.edu.pl (D.P.); miroslaw.bocian@pwr.edu.pl (M.B.); 3 Institute of Mechanics and Printing, Faculty of Production Engineering, Warsaw University of Technology, Narbutta 85, 02-524 Warsaw, Poland; granada@pompy.pl (M.B.); mariusz.magier@pw.edu.pl (M.M.); 4 Wroclaw Center for Technology Transfer of Wroclaw University of Science and Technology, Smoluchowskiego 28, 50-372 Wroclaw, Poland; jan.koch@pwr.edu.pl

**Keywords:** ballistic resistance, composite materials, dynamic loads, computational modelling, squeeze casting

## Abstract

The paper presents the results of studies on the effects of shooting composite materials produced by pressure infiltration with the EN AW-7075 alloy as a matrix and reinforcement in the form of preforms made of α-Al_2_O_3_ particles. Composite materials were made with two reinforcement contents (i.e., 30% and 40% vol. of α-Al_2_O_3_ particles). The composites produced in the form of 12 mm thick plates were subjected to impact loads from a 7.62 × 39 FMJ M43 projectile fired from a Kalashnikov. The samples of composites with different contents of strengthening particles were subjected to detailed microscopic examination to determine the mechanism of destruction. The effect of a projectile impact on the microstructure of the material within the perforation holes was identified. There were radial cracks found around the puncture holes and brittle fragmentation of the front surfaces of the specimens. The change in the volume of the reinforcement significantly affected the inlet, puncture and outlet diameters. The observations confirmed that brittle cracking dominated the destruction mechanism and the crack propagation front ran mainly in the matrix material and along the boundaries of the α-Al_2_O_3_ particles. In turn, numerical tests were conducted to describe the physical phenomena occurring due to the erosion of a projectile hitting a composite casing. They were performed with the use of the ABAQUS program. Based on constitutive models, the material constants developed from the identification of material properties were modelled and the finite element was generated from homogenization in the form of a representative volume element (RVE). The results of microscopic investigations of the destruction mechanism and numerical investigations were combined. The conducted tests and analyses shed light on the application possibilities of aluminium composites reinforced with Al_2_O_3_ particles in the construction of add-on-armour protective structures.

## 1. Introduction

In the previous engineering applications of materials resistant to impact (ballistic impact), composite materials occupy a significant position [1,2,3,4,5]. In mathematical terms, the phenomenon of energy absorption by materials subjected to impact at ballistic velocities is a complex process [6,7,8,9]. Efforts to understand the physical processes are directed towards searching for new material solutions, the mechanical properties of which should exceed those of the traditional material. Such materials include ceramics, polymers, and composites, which are now a new option [10,11,12,13,14]. Each material and composite should meet the need for continuous improvement in the development of missiles and weapons, and, under specific conditions, new applications [15,16,17].

Alternative materials can be composites with a metal matrix reinforced with ceramic particles or fibres, the so-called cermets (metal matrix composite—MMC) [18,19,20]. These materials mainly feature a relatively low density (3.95 g/cm^3^ for aluminium oxide, approximately 2.7 g/cm^3^ for aluminium) and good mechanical properties, such as impact strength, tensile strength, yield strength or elongation [21,22,23].

Cermets can largely replace the standard material in the form of classical ballistic ceramics and the works conducted [24,25,26,27] are aimed at searching for proper structures which absorb the energy of the ballistic impact. The metal matrix composite is mainly used in armouring applications to prevent perforation. Under dynamic loading conditions, such as missile-to-cermet contact, the material is designed to maintain high-stress deformation. 

In one study [28], the authors investigated the interaction between a 7.62 × 51 mm armour-piercing (AP) projectile and a composite shield (MMC) with 20% SiC reinforcement. The authors focused mainly on post-impact crack testing, where they estimated the deformation of the matrix alloy grain and the rate of deformation due to contact with the SiC particle reinforcement. Another study [29] concerned ballistic loading with a 7.62 mm AP projectile with an impact velocity of 710 m/s of MMCs reinforced with 15%, 30% and 45% SiC particles. Wear and damage mechanisms were evaluated using SEM and optical microscopy, which investigated the projectile tips and hole surfaces resulting from high-velocity impact. Carbajal et al. [30] narrowed their considerations only to the development of a model using the finite element method for a 7.62 × 39 mm projectile with a mild steel core. Experimental studies using the Taylor test validated the results.

The behaviour of ceramic tiles and tiles made of cermet was interestingly compared in another study [31]. In the preliminary research, the authors observed differences in the patterns of macro-fractures occurring during the impact of ceramic and cermet tiles with different levels of closure. The primary experimental research was focused on determining the effect of pressure on model ceramic materials and cermets. The mechanisms of deformation and cracking were analysed both in quasi-static tests and in dynamic loads. Loiseau et al. [32] studied the ballistic response of Cr/CrS cermets as a function of chromium metal content under two ballistic test conditions. In the first experimental set, thin cermet samples (thickness 3.5 mm) were wholly enclosed in a metal anvil and hit with small steel balls at a speed of 1460 m/s. Macro-scale observation of crater formation, fragmentation and cracking of recovered samples, as well as electron microscopy of the impact zone, revealed material-dependent changes in response to damage. In the second experimental set, Cr/CrS cermet discs with a constant surface density but with different compositions were employed for veneering with a long cylinder of high-density polyethylene. The 2.85 g fragment simulating projectile (FSP) impacted the cermet discs at a velocity of 1715 m/s, and the residual depth of polyethylene penetration was measured.

Several other studies [33,34,35] refer to the understanding of the dynamic damage of aluminium composite reinforced with Al_2_O_3_ particles based on either a clear understanding of homogeneous deformations of the composite and matrix material in dynamic conditions or microscopic observations of the failure mode. They also address the mechanics of response to a high-strain ratio and the determination of the mechanism associated with dynamic failure in tension.

The mechanisms of friction and damage to MMC armouring systems have been described by several studies [36,37,38]. Newly developed metal–ceramic composites with the aluminium matrix for indirect ammunition 5.56 × 45 SS109 shooting were studied. The composite material produced by the squeeze casting method was based on AC-44200 alloy strengthened with preforms from Al_2_O_3_ particles [39]. Metal matrix composites with 20% and 40% vol. of Al_2_O_3_ particles were analysed in relation to the material of the non-reinforced matrix. The results obtained from the modelled ballistic impact were compared to the actual damage. Due to the introduction of new solutions in innovative materials, the results were collated with the solutions based on corundum ceramics and the traditional armour plate, in order to assess that the optimization of adoption possibilities in composite ballistic shields.

In the present paper, the authors focused on samples made of MMCs materials with 30% and 40% vol. of Al_2_O_3_ particles. The samples were shot at using a 7.62 × 39 full metal jacket (FMJ) M43 projectile fired from a Kalashnikov. The aim of the present study was to investigate the impact resistance of the analysed cermets. In the experiment, the absorbed impact energy and residual energy were estimated from the impact velocity and the outlet velocity after the samples were shot. Next, metallographic investigations were conducted using SEM of the destruction mechanisms in relation to samples with 30% and 40% reinforcement with Al_2_O_3_ particles by volume. Using numerical simulations, it was shown which mechanisms influence the process of impact energy dissipation. The obtained results were compared with experimental results. On this basis, conclusions on the implementation of cermets in ballistic applications were formulated. 

## 2. Object and Methodology of Research and Test Bench

### 2.1. Composite Test Materials

The shooting tests were performed on metal composite materials produced by the infiltration of porous ceramic preforms (squeeze casting) using the casting method of pressing from a liquid state. In the first stage of material production, the preforms with a 30% and 40% vol. of Al_2_O_3_ particles were prepared, in which connections were made using a hydrated solution of sodium silicate Na_2_O·nSiO_2_·xH_2_O (n, x—stoichiometric coefficients), cured with CO_2_, and stabilized at high temperatures (>960 °C).

Preforms were made in the form of 62 × 42 × 12 mm rectangular tiles. The preforms heated to 700 °C were placed in a durable steel form of *ϕ* = 100 mm circular cross-section and flooded with a liquid matrix alloy for infiltration purposes. Then, a pressure of 90 to 110 MPa was exerted through a pressing stamp, causing the warp to infiltrate into the free spaces of the preform. In this way, samples with the base material AW-7075 (S1), samples with 30% Al_2_O_3_ particle reinforcement (S2) and samples with 40% particle reinforcement (S3) were prepared. Table 1 and Table 2 present the chemical specification of the matrix material and reinforcing particles. Tests confirming the compliance of the chemical composition with the EN 573-1 standard of EN AW-7075 material supplied by the manufacturer were performed using an S1 MiniLab 150 analyser.

Basic mechanical properties of the manufactured materials were subjected to testing the results of which are presented in Table 3.

### 2.2. Ballistic Test Bench

The firing of samples was performed on a ballistic test bench using a 7.62 × 39 FMJ M43 projectile fired from a Kalashnikov (AK-47). Each shot was recorded by measuring instruments such as a Radar Doppler Waibel SL-525P and Chronograph CED Millennium. Only those shots for which the muzzle velocity parameters did not fall within the range of 715 ± 10 m/s were rejected. Thus, the ballistic test was performed under the guidelines of the European standard EN 1522 [40]. 

**Figure 1:**
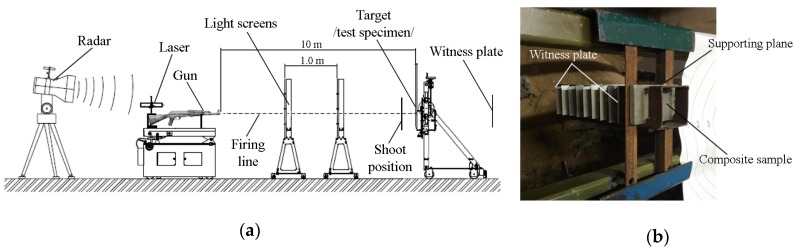
A bench for shooting of composite materials firing station: (**a**) Schematic diagram according to EN 1522 standard; (**b**) method for the attachment of samples.

The ballistic tests were done on a bench consisting of a tunnel-shaped rig equipped with partitions allowing the assembly of composite samples and witness-type sheets. The rig construction enabled stable seating of composite specimens with two-point support on the front surfaces of the brackets. A set of 0.3 mm thick aluminium witness plates were installed behind the composite sample at distances of 20 mm. The plates constituted the basis for the assessment of the casing puncture [41]. Figure 1 displays the bench with a mounted specimen prior to firing. 

### 2.3. Preliminary Studies on the Projectile Structure 

A detailed chemical analysis of the core, jacket and lead jacket of the projectile was conducted, together with an evaluation of the microstructure in order to thoroughly examine the microstructural changes in the composite materials under the dynamic impact of the 7.62 × 39 FMJ M43 projectile.

**Figure 2:**
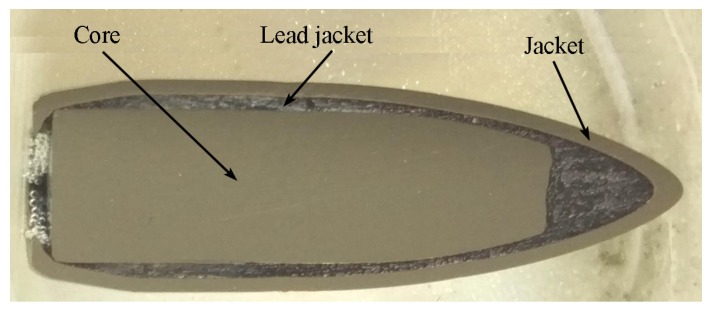
Longitudinal section of the 7.62 × 39 FMJ M43 projectile.

Figure 2 illustrates the general structure of the projectile in the example of the longitudinal cross-section.

Spectrometric examinations of the chemical composition of both the core and the jacket material indicated low carbon steel (see Table 4). 

**Figure 3:**
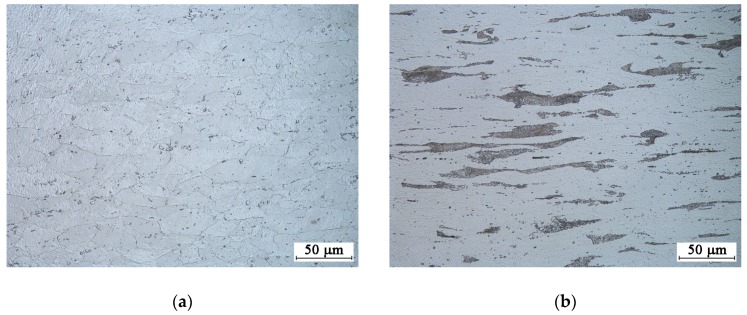
A material microstructure of the 7.62 × 39 FMJ M43 projectile: (**a**) Core; (**b**) jacket. Trawino—5% HNO_3_.

Meanwhile, microscopic examinations confirmed the ferritic structure of the projectile jacket (see Figure 3a) and the ferritic-perlitic structure of the projectile core with a different intensity of the perlite band distribution (see Figure 3b).

**Figure 4:**
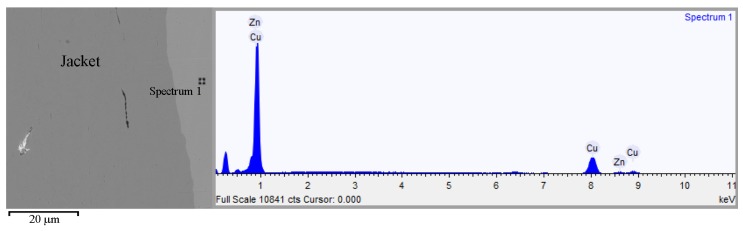
An EDS analysis of a material layer on a jacket surface of the 7.62 × 39 FMJ M43 projectile.

Moreover, the Cu-Zn alloy layer with chemical specification confirmed by EDS tests was additionally found on the surface of the jacket material in the place shown in Figure 4 (Spectrum 1), and the results of the determination are given in Table 5. In the EDS tests of the Pb lead jacket filling the space between the jacket and the core, individual Sb antimony precipitates were also present. 

### 2.4. Metallographic Tests

The surfaces and cross-sections of the shot composite samples were subjected to macro and microscopic examination. Microstructure analysis after firing was performed using light microscopy with a Nikon Eclipse MA200 microscope and SEM on a Hitachi TM-3000 microscope equipped with an EDS system. The observations were made on surfaces of the hole created after the projectile’s passage, on the surfaces of radial and internal cracks. Moreover, analysis of microstructural changes in the areas directly affected by the projectile’s impact occurring inside the material directly below the penetration surface was performed.

### 2.5. Numerical Analysis

**Figure 5:**
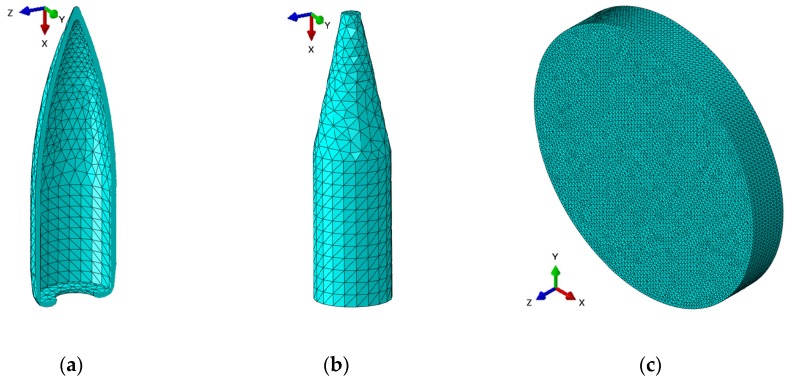
Numerical models of a projectile and a sample: (**a**) Jacket; (**b**) core; (**c**) composite sample.

The numerical models of the projectile and the tested samples were discredited with Tet-type solid elements (see Figure 5), where each item was 1.0 mm in size (see Table 6). 

Numerical analysis of the perforation of metal composite materials was performed in ABAQUS using the Explicit method. An initial velocity of 715 m/s and a mass of 7.9 g for the projectile, including 3.6 g for the steel core, were assumed. The projectile geometry was taken from a previous study [42], while some parameters of the core damage from other work [43] were taken into account.

Assessing the reaction of a material to high velocity impact includes considering the effects of deformation, strain rate and temperature. Therefore, the Johnson–Cook (J-C) elastic-plastic model was adopted for mathematical description, including the rheological properties of materials [44,45,46,47,48].

(1) $$\sigma =\left(A+B\varepsilon_{p}^{n}\right)\cdot (1+C\ln{\dot{\varepsilon }}^{*})\cdot \left[1-{\left(T^{*}\right)}^{m}\right]$$

where *σ* is the equivalent stress, and *ε_p_* is the equivalent plastic strain. The material constants are *A*, *B*, *m*, *n* and *C*. *A* is the yield stress of the material under reference conditions, *B* is the strain hardening constant, *n* is the strain hardening coefficient, *C* is the strengthening coefficient of strain rate, ${\dot{\varepsilon }}^{*}$ is the dimensionless strain rate and T^*^ is the non-dimensional temperature [49].

Non-dimensional temperature is defined as:

(2) $$T^{*}=\frac{T-T_{room}}{T_{melt}-T_{room}}$$

where *T* is the actual temperature based on plastic work, *T_room_* is the room temperature and *T_mel_*_t_ is the melt point temperature. Required material properties were taken from experimental static (tensile tests) and dynamic (split Hopkinson pressure bar (SHPB) tests [50] performed using samples obtained from I-beam material.

The J-C destruction model associated with the accumulation of plastic deformation, described in the form presented below, was adopted for the jacket and the projectile core:

(3) $$d=\sum \frac{\Delta \varepsilon_{p}}{\varepsilon_{f}}$$

where Δ*ε_p_* is an increment of the equivalent plastic strain and *ε_f_* is the equivalent plastic deformation until failure. Therefore, the J-C model was supplemented with a model of destruction in the form:

(4) $$\varepsilon_{f}=\left(d_{1}+d_{2}e^{d_{3}\sigma^{*}}\right)\cdot \left(1+d_{4}\ln{\dot{\varepsilon }}^{*}\right)\cdot \left(1+d_{5}T^{*}\right)$$

where *d*_1_ to *d*_5_ are failure parameters measured at or below the transition temperature, $\sigma^{*}=p/\sigma_{eff}$ is a dimensionless ratio expressed as the pressure *p*, *σ_eff_* is the effective stress and ${\dot{\varepsilon }}^{*}={\dot{\bar{\varepsilon }}}^{p}/{\dot{\varepsilon }}_{0}$ is expressed as a ratio equivalent to the plastic strain rate ${\dot{\bar{\varepsilon }}}^{p}$ and reference strain rate ${\dot{\varepsilon }}_{0}$.

The dynamic model of steel core damage is based on effective plastic displacement, which is defined by the equation:

(5) $${\dot{\bar{u}}}^{p}=l{\dot{\bar{\varepsilon }}}^{p}$$

where *l* is the characteristic length of the element. 

The parameter σ^*^ is a measure of the triaxial stress state prevailing in the material and the parameters *d*_1_ to *d*_5_ are dominant over the others. The parameter *d*_1_ defines the asymptotic deformation under failure conditions when the stress tends to stretch hydrostatically. It is characteristic of the J-C failure model that after the element is damaged, its stiffness and strength tend to zero automatically. The damage occurs when the failure parameter d reaches 1.

A hybrid method of combining FEM and SPH algorithms was applied to avoid disturbances in the algorithm related to mass increase (Δm). After exceeding a given destructive strain, the FEM element was converted to the SPH point having *i*-th mass (*m_i_*), so that the mass in the system remained constant [50,51,52,53].

Table 7 summarizes the values of the mechanical properties of the tested composites and projectile that were assumed for numerical simulations.

The boundary conditions were set in such a way that the numerical model reflected, as much as possible, the system features during experimental research. The rotational speed was omitted due to its negligible influence on the kinetic energy dissipation. There was a ballistic shield fixed around the perimeter. The restraint of the shield blocked translations and rotations in the three *X*, *Y* and *Z* axes. The analyses used the default contact model based on the “punishment function” method [45]. 

## 3. Results and Discussion

### 3.1. Analysis of the Shooting Samples

After the shooting of the S1, S2 and S3 samples, a detailed analysis was performed to assess the energy consumption and determine the residual speed. The ballistic limit for the samples was defined from $v_{50}.$ This velocity is identified as an average of the equal number of the highest partial penetration velocities and the lowest total penetration velocities for a given combination of projectile and target, which occur within the specified velocity range [53]. The ballistic speed limit was set based on the projectile impact velocity with a 50% possibility of total penetration of the samples. In the case of energy absorption, the difference in the kinetic energy of the projectiles was calculated as the energy absorbed by the composite plate. Assuming that the projectiles are not deformable, the following equations were previously used [54]:

(6) $$v_{50}=\sqrt{\left(v_{i}^{2}-v_{r}^{2}\right)}$$

(7) $$E_{ab}=\frac{1}{2}m\left(v_{i}^{2}-v_{r}^{2}\right)$$

where m is the mass of the projectile in kg and $v_{50}$ are the impact and residual velocities of the projectile in m/s, respectively.

It was also presumed that the velocity of the projectiles is constant from the beginning of the propulsion until the moment they hit the samples, and their energy loss is proportional to the energy absorbed by the samples. In terms of the relationship between impact and residual velocities, it was observed that an increase in initial velocity caused an increase in the residual velocity for all thicknesses. The impact velocity at zero residual velocity corresponds to $v_{50}$.

**Figure 6:**
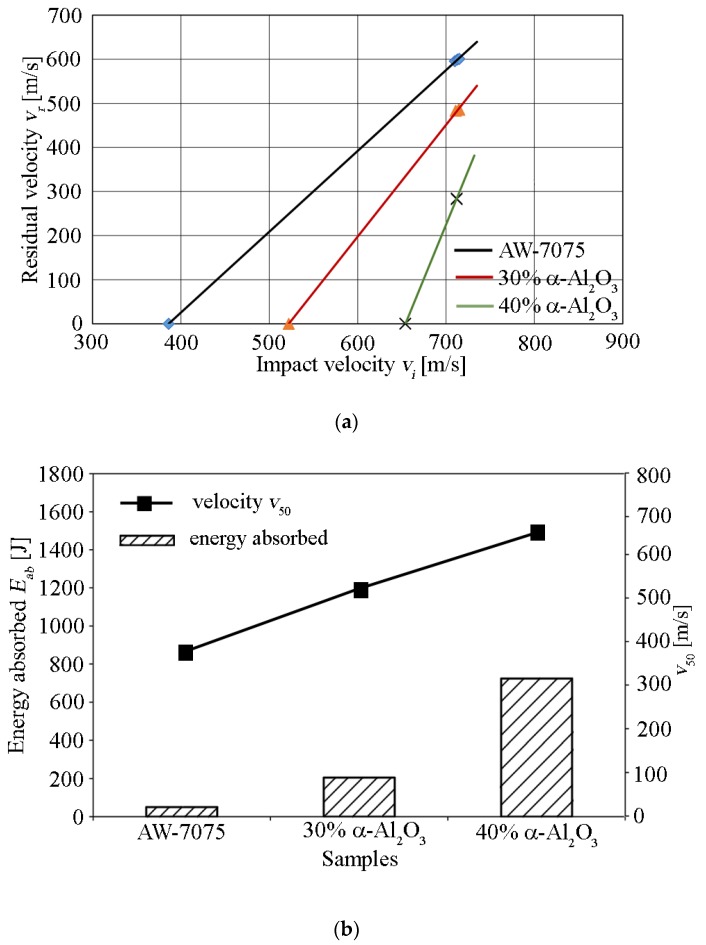
Evaluation of tested samples during ballistic impact: (**a**) Residual velocity as a function of impact velocity for composites samples; (**b**) energy absorption and ballistic limit $v_{50}$ for analysed samples.

The impact and absorbed energy are the two main parameters used to assess the ballistic impact properties of composite materials. The ballistic test was performed on three types of samples (S1 to S3) in a series of three each. Figure 6b shows the $v_{50}$ velocity and energy absorption of the test samples at impact speeds from 710 to 716 m/s. The highest velocity $v_{50}$ and energy absorption was recorded for S3, at 654 m/s and 724 J, respectively. Sample S2 absorbed 205 J and the velocity $v_{50}$ was 522 m/s. Sample S1 was the base material (i.e., the matrix), which proved to be the weakest, since the velocity $v_{50}$ was only 386 m/s, while the energy absorption was estimated at 51 J.

It should be emphasized that that elements such as the geometry of the projectile and target, velocity and energy of the impact, angle of the projectile hitting the target and properties of the target material and the projectile, including density, influence those two parameters [55].

**Figure 7:**
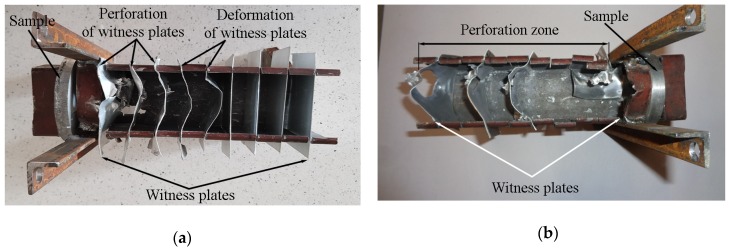
Damaged aluminium witness plates after shooting of the tiles of composite material with: (**a**) Sample S3; (**b**) sample S2.

The organoleptic evaluation of the witness plates showed that sample S3 absorbed such a high amount of impact energy that on the fifth witness plate, the projectile was braked (see Figure 7a). The first three plates were perforated, while the remaining two plates were only deformed. The shooting of sample S2 resulted in complete perforations of the witness plates (see Figure 7b).

By the size of holes, it can be said that the projectile after firing experienced tumbling, which has a positive effect on its residual energy reduction. It was also found that the projectile’s jacket and lead jacket were torn off in the core already at the stage of its penetration into the sample.

### 3.2. Macroscopic Analysis of the Shot Samples

**Figure 8:**
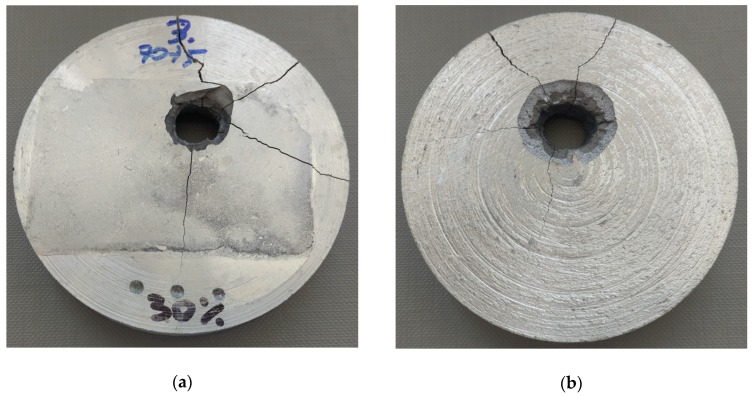
View of a sample S2 after firing with the 7.62 × 39 mm projectile: (**a**) Projectile’s inlet surface; (**b**) projectile’s outlet surface.

**Figure 9:**
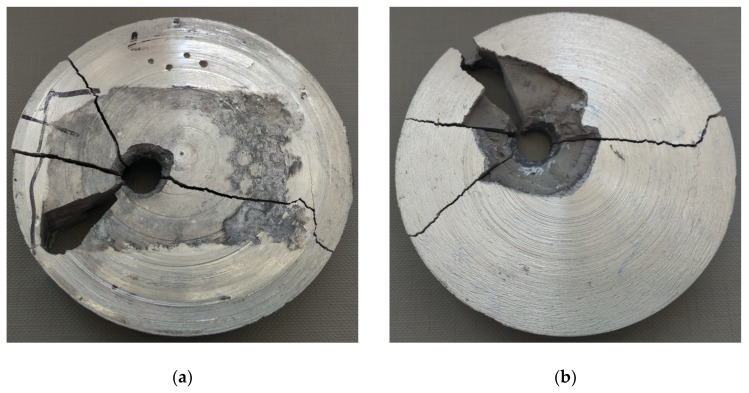
View of a sample S3 after firing with the 7.62 × 39 mm projectile: (**a**) Projectile’s inlet surface; (**b**) projectile’s outlet surface.

The firing of the projectiles resulted in the perforation of the materials tested. The holes have been depicted with views of the front and side surfaces of the samples. In the samples reinforced with 30% (S2) and 40% (S3) of α-Al_2_O_3_ particles, a projectile hole was created with an inlet diameter of 8.6 to 9.5 mm and 8 to 8.5 mm. Brittle cracks propagated radially from the crater. Figure 8 and Figure 9 present a view of the surface of the samples from the projectile inlet and outlet side. Partial fragmentation of the material can be observed on the surface of the cracks.

**Figure 10:**
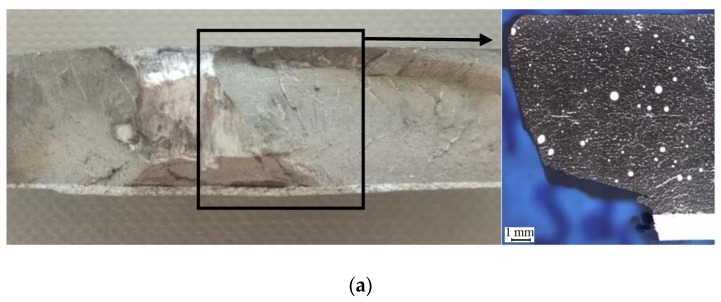

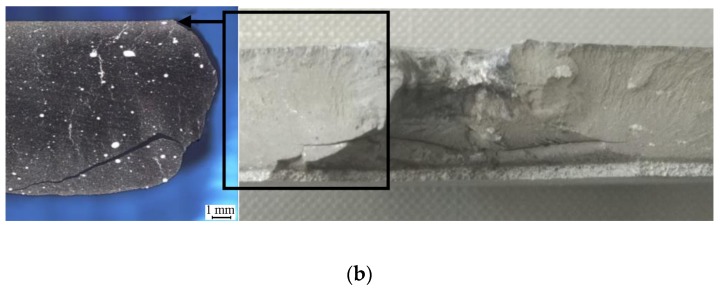
View of a projectile passage surface in the analysed samples: (**a**) In the material of sample S2 after firing; (**b**) in material of sample S3 after firing.

In the resulting perforation hole, several zones with different morphology, which are clearly visible in the cross-section illustrated in Figure 10, can be distinguished. A characteristic brittle splintering zone of the material formed around the resulting crater was created from the side of the projectile inlet. A change in the diameter of the chipping, ranging from 15 to 18 mm in materials with 30% vol. of α-Al_2_O_3_ particles and 16 to 21 mm in materials with 40% vol., was noticeable and is typical behaviour of brittle materials [56,57]. From the projectile’s outlet side, a local fragmentation took place over the entire thickness, and thereby a through-hole was made. However, this fragmentation can be described as a spalling phenomenon, which is characteristic of brittle materials. The formation of the so-called Hertz cone effect caused numerous stratifications in the material of a brittle fracture character running transverse to the direction of the puncture. From the side of the projectile’s entry, due to radial forces, a partial brittle radial crack occurred combined with a brittle rebound of material fragments.

**Figure 11:**
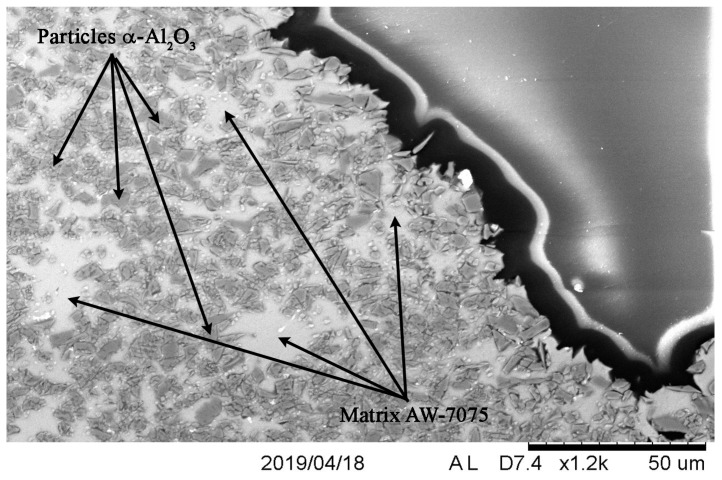
Edge formed by brittle cracking in the front surface of a sample S2.

Microscopic analysis conducted on the cross-section in the chipping zone of the material on the front of the sample confirmed the tendency towards brittle cracking. Figure 11 shows the exemplary surface formed by the separation of the material layer. In this case, the crack trajectory mainly occurred along the boundaries of α-Al_2_O_3_ particles with their clear separation from the matrix material. Observations of the material under the crack surface indicated no significant changes in the structure, but only a slight displacement of particles. It resulted in a slight fragmentation involving small portions of the material being torn off and a small amount of cracking of strengthening particles.

**Figure 12:**
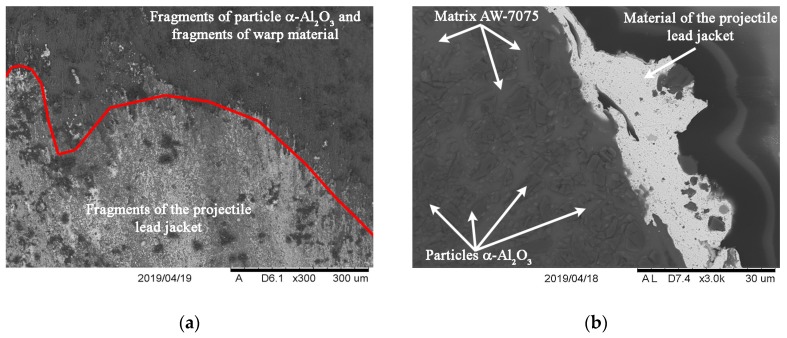
A crater surface in the projectile inlet area. A sample S3: (**a**) Surface view; (**b**) cross-section.

Then, the projectile inlet area was distinguished in the puncture crater. The surface, which blocks the projectile from entering, was significantly deformed plastically. Due to the high-impact energy, the temperature increased and the lead jacket of the projectile melted. The lead material showed excellent adhesion to the surface of the composite material. The thickness of the lead layer, depending on the location, ranged from 3 μm on flat surfaces to as much as 30 µm in grooves forming cavities on the penetration surface. Numerous fragments of the projectile jacket, particles, fragments of crushed α-Al_2_O_3_ particles and fragments of warp material were found in the adhesively-bonded lead layer (see Figure 12).

The analysis of the crater surface in the projectile inlet zone indicated radially-distributed streams of the clotted lead material of the projectile lead jacket. The microstructures of melted and clotted material on the crater surface were arranged in the direction of radial forces in the form of continuous strands or frozen droplets with a relatively spherical shape (see Figure 12a). 

**Figure 13:**
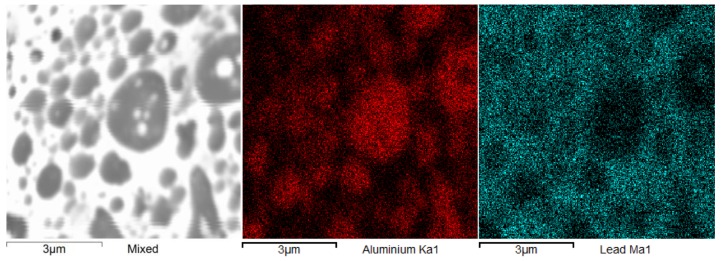
EDS: Map of elemental distribution on the crater surface of a sample S2.

Figure 13 presents the results of the EDS identification analysis together with the assessment of the chemical composition of the surface layer of crater.

Spherical clearances with smooth edges were formed in the layer of clotted lead. This suggests that at accompanying temperature conditions, the Pb material shows good wettability for the surface of the composite material within the impact area. During solidification, with significant contraction in the liquid state, it deflected the surface of the composite material. The molten lead deposited on the surface appeared as a continuous mesh.

**Figure 14:**
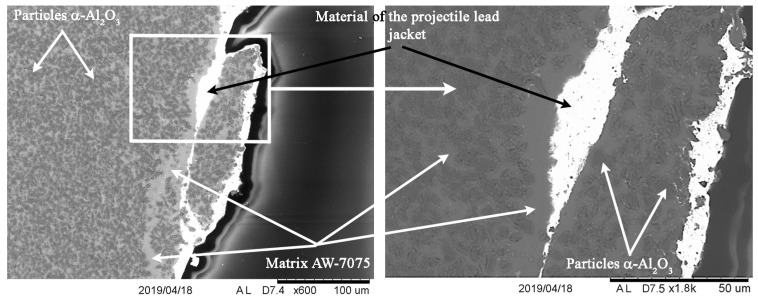
A surface of the crater interior in a projectile hole. A sample S3.

**Figure 15:**
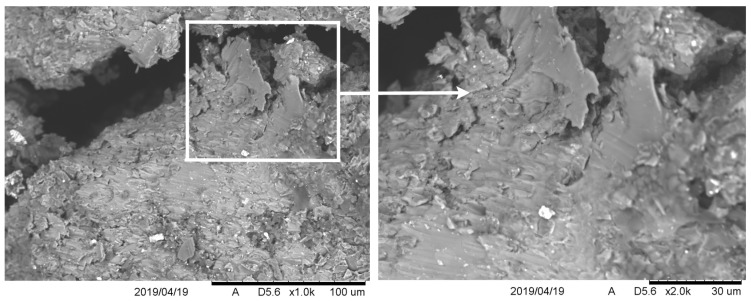
SEM: Crater surface in a projectile inlet area. A sample S2.

On the surface, in the zone called the puncture surface, the material structure indicated the occurrence of the plastic flow of the material with a slight fragmentation. The latter caused partial separation and bending of the material, leading to the formation of a characteristic scaly texture on the surface. The material of the Pb projectile jacket tightly adhered to the surface of the exfoliated material (see Figure 14). Its surface had clear traces of projectile core interaction, which can be classified as characteristic of surfaces subjected to shear forces (see Figure 15). Penetration of the molten lead Pb into the spaces formed by the shells deflected from the crater surface was also found. After solidification, lead maintained a strong adhesive bond with the surfaces of the composite material and peeled shells.

**Figure 16:**
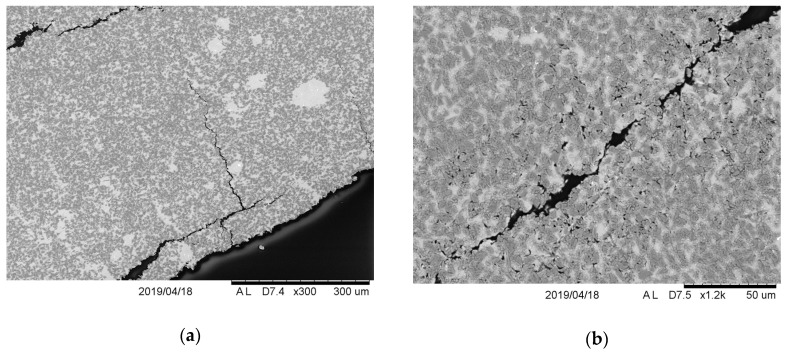
Cracks and delamination in the material S2 from a projectile outlet side visible in the cross section: (**a**) Fragmentation; (**b**) porosity in the fracture area.

A brittle fragmentation of the material at significant angles occurred in the next projectile impact zone on the outside. Due to radial forces, crushed fragments were pushed outwards, which caused their wedging with the adjacent crater surfaces. Hence, friction on the surfaces of the fragments to be torn off with the crater surfaces took place, along with significant shear forces. The described dynamic interaction in the material structure caused not only traces of particle displacement in the matrix material, but also cracks and separation of particles from the matrix were found (see Figure 16).

### 3.3. Analysis of Numerical Results

**Figure 17:**
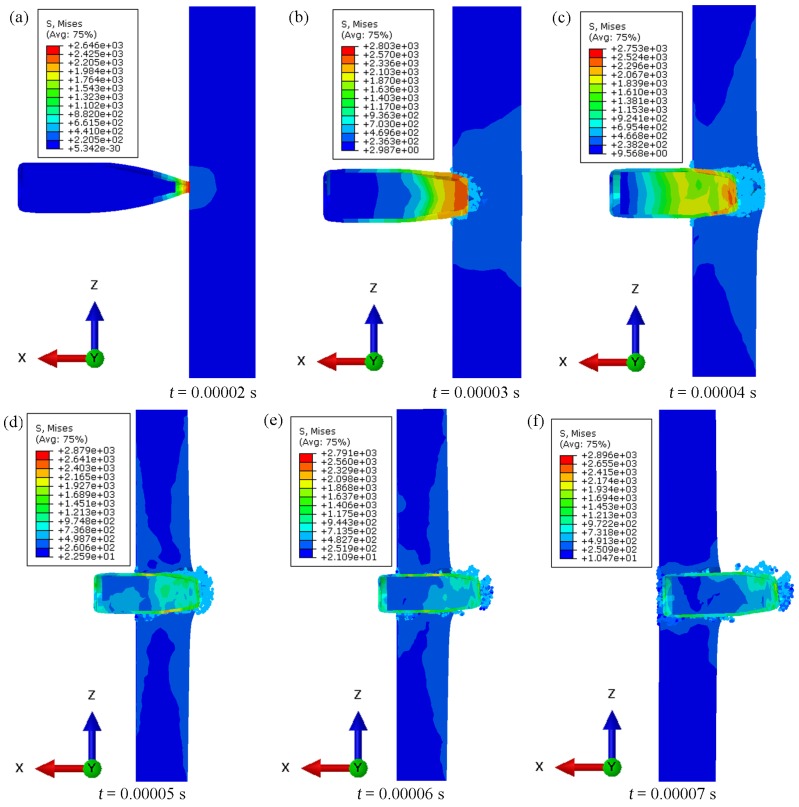
Summary of individual penetration time steps in numerical simulation for a sample S2.

**Figure 18:**
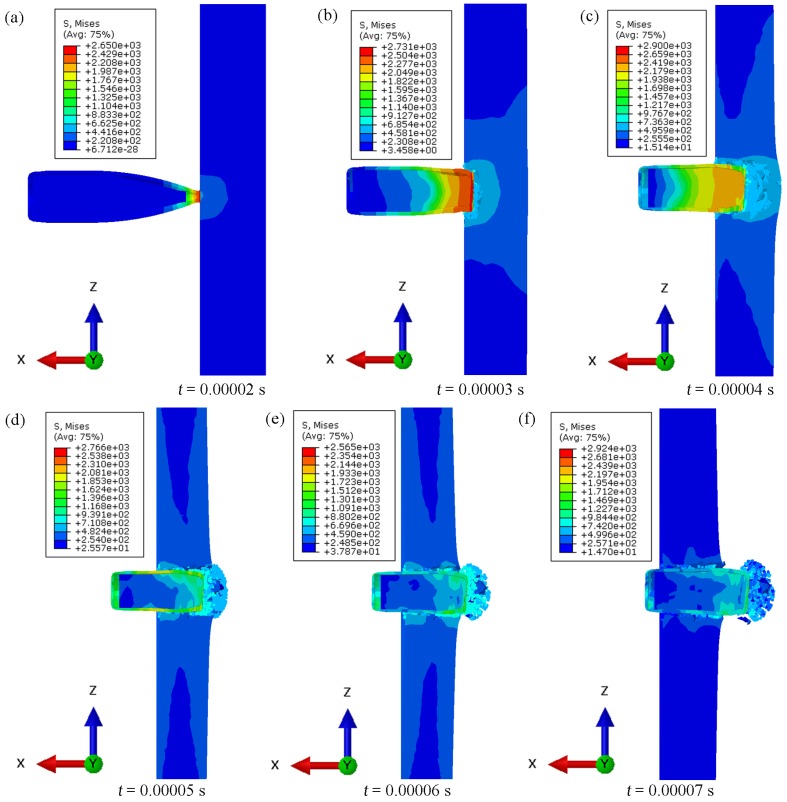
Summary of individual penetration time steps in numerical simulation for a sample S3.

Figure 17 and Figure 18 show the results of the time steps for the penetration of samples S2 and S3, analysed based on the numerical experiments conducted under the conditions described in Section 2.5.

The results obtained for samples reinforced with 30% and 40% reinforcement of α-Al_2_O_3_ particles differed in the impact energy scattering mechanism. The simulation for the material with 30% reinforcement (Figure 17) was characterized by a lower deformation of the impacting projectile. In individual time steps, the stress layers assumed maximum values in the first stage (i.e., the projectile’s penetration and its radial dispersion and erosion in the composite material of the sample under consideration). After exceeding the time value of 0.00004 s, the stress layers of the projectile were considerably reduced as the projectile speed significantly slowed down. It follows that the material was already so weakened by chunking and the formation of fatigue gaps that it did not provide significant resistance. The geometry of the projectile was not severely deformed and it can only be concluded that mainly the jacket and lead jacket were destroyed (i.e., torn off the core). The simulation for the material with 40% reinforcement (Figure 18) showed a higher tendency to increase the projectile’s geometric deformation. For the same time steps, the stress values were much higher. The material weakening was also observed in the simulations, after exceeding the same time step as for samples with 30% reinforcement. The difference is in the higher proportion of the chunking of the sample material layers from the projectile outlet side of the sample. The projectile’s geometry was more deformed by 40% reinforcement. The absorption of the impact energy was greater, which manifested in the more substantial brittleness of the sample tested. There were more chipped off parts of the composite material on the front of the projectile.

**Figure 19:**
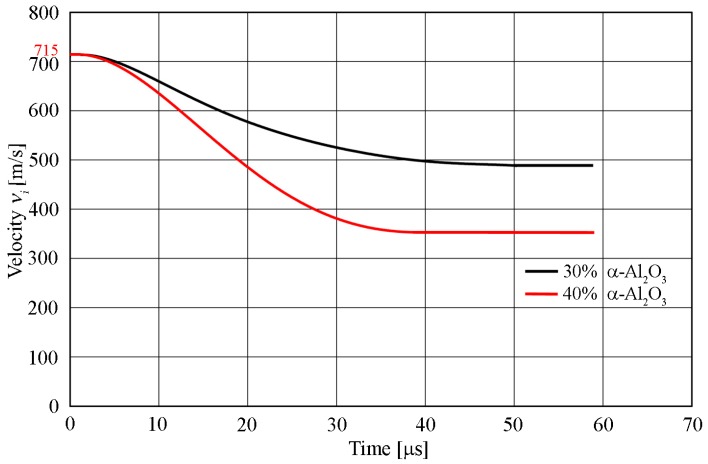
Diagram of a projectile velocity for the two types of material obtained from the numerical simulation.

Another aspect of the numerical analysis was to determine the changes in the velocity of the striking projectile in individual time steps. The following velocity values after the projectile passed, for which the initial velocity was 715 m/s, which corresponds to the real conditions of the experiment, are shown in Figure 19. The projectile left the composite material with 30% α-Al_2_O_3_ particle reinforcement at a speed of approximately 495 m/s. This sample was able to decelerate the projectile by 30.8%, which provided an estimated speed of 220 m/s. The sample of AW-7075 with 40% of α-Al_2_O_3_ particles reduced the projectile velocity by 49% (i.e., the projectile velocity at the outlet of the sample was about 350 m/s). It was noted that the reinforcement of the base material with a higher volume of α-Al_2_O_3_ particles showed a better ballistic performance. 

**Figure 20:**
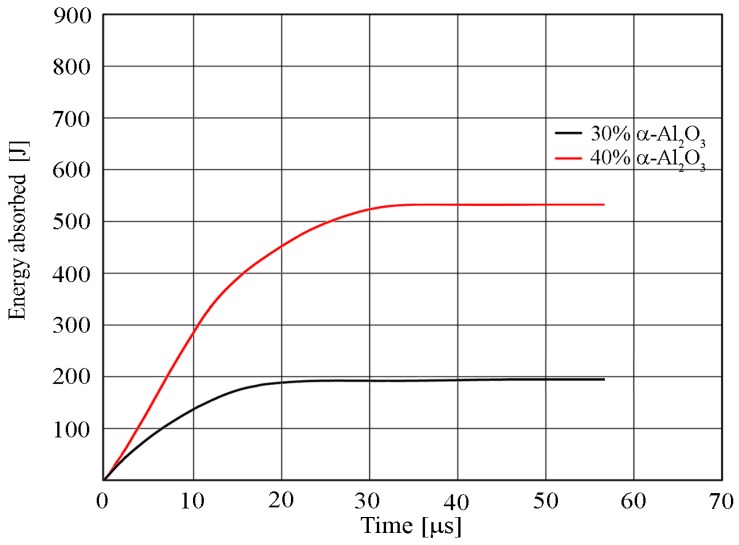
Diagram of the impact energy absorption by the projectile–material AW-7075 reinforced with α-Al_2_O_3_ particles obtained from numerical simulation.

Figure 20 presents the changes in the energy absorbed by the projectile–material system loaded with ballistic impact. The sample with 30% reinforcement of α-Al_2_O_3_ particles showed a reduction of initial kinetic energy of approximately 192 J, while for the sample with 40% reinforcement of α-Al_2_O_3_ particles, a reduction in kinetic impact energy of about 527 J was observed.

### 3.4. Comparison of Experimental and Numerical Results

**Figure 21:**
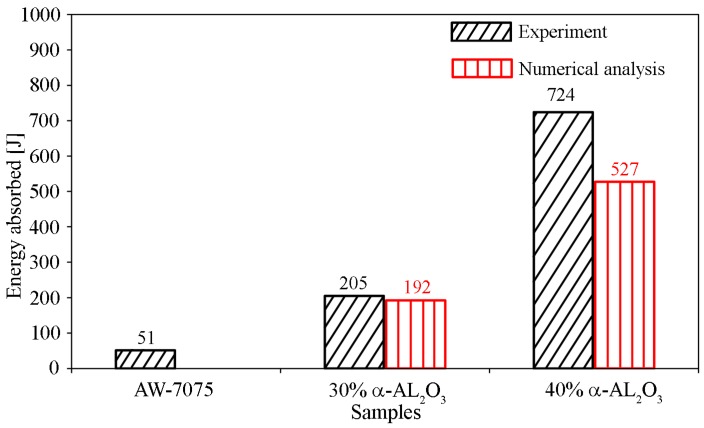
Comparison of results of absorbed impact energy of the projectile–shield system in the material under analysis.

In the qualitative evaluation of the results obtained from the numerical analysis, they were compared with experimental results. The comparison of the absorbed kinetic impact energy in the projectile–shield system is shown in Figure 21. High compliance was obtained for sample S2, with a 6.3% difference between the ballistic experiment and the numerical simulation. The result for sample S3 already constituted a much bigger dispersion, with a difference of 27.2%. With a match assumed at the level of 30% [58], the result is satisfactory. This difference stems mainly from the fact that the simulated system removed the finite elements after exceeding the limit parameters. Such a situation is a feature of the finite element method. 

**Figure 22:**
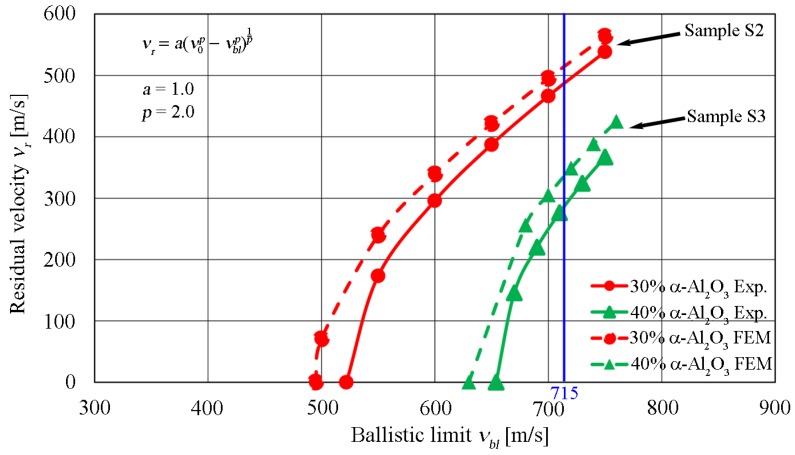
Diagrams of residual velocity for the analysed samples S2 and S3.

The obtained comparative results on the example of residual velocity juxtaposition (see Figure 22) indicated satisfactory development of the numerical models.

Based on the analysis of velocities obtained in the experiment and numerical simulation, the residual velocity, $v_{r}$, was estimated at 488 m/s for the experiment. In the FEM numerical simulation, this velocity was estimated at 502 m/s for sample S2 (30% vol. of α-Al_2_O_3_ particle reinforcement) at the impact velocity $v_{0}$ of 715 m/s. For sample S3 (40% vol. of α-Al_2_O_3_ particle reinforcement), the residual velocity was estimated to be approximately 350 m/s for the FEM simulation. From the experiment, the residual velocity was estimated at 287 m/s. The ballistic limit $v_{bl}$ for sample S2 is approximately 522 m/s in the experiment, while in the FEM analysis, it was approximately 495 m/s. Sample S3 had a ballistic limit $v_{bl}$ of about 654 m/s in the experiment and an estimated 630 m/s in the FEM analysis. The difference in $v_{bl}$ results for S2 was 5.2% and 3.7% for S3.

## 4. Conclusions

The application of the EN AW-7075 alloy, classified as a plastic processing material in the casting of composite materials, influenced the reduction of the kinetic energy impact of the 7.62 × 39 FMJ M43 positively. The advantages resulting from the possibility of obtaining significant plastic deformation of the matrix, combined with the use of hard non-deformable ceramic reinforcing elements, had a significant positive effect on the mechanism creating scrap during ballistic tests. The application of ceramic α-Al_2_O_3_ particles with a volume of 30% and 40% in the tested composite materials, while maintaining the ability for plastic deformation, allowed for adequate absorption of the kinetic energy of the projectile. 

At the same time, during the dynamic projectile impact, the presence of hard non-deformable particles increased the process of the missile speed deceleration. As shown in Figure 7, after the shooting of a material 30% vol. of α-Al_2_O_3_, the projectile penetrated all witness plates, while in the case of a material 40% vol. of α-Al_2_O_3_, it was braked on the fifth septum of the witness plate.

The compared matrix material (i.e., sample S1) was the least effective. This shows that only materials with different types of particle additions can meet the ballistic limit and can be used as an alternative to steel or older generation composite materials.

Supporting the ballistic analysis with numerical simulations is highly advisable, as it extends the possibilities of adopting known constitutive models by modifying them with a group of new materials.

In summary, the presented analysis of the dispersion of the impact energy from the 7.62 × 39 FMJ M43 projectile fired into a newly developed material for impact energy absorbers is a promising direction. Searching for optimal solutions to reduce the weight of the ballistic system and its increased efficiency is the main task of designers of modern ballistic shields. 

## Figures and Tables

**Table 1:** Chemical composition of base material EN AW-7075.

Element [% Wt]	Zn	Mg	Cu	Fe	Si	Mn	Cr	Zr	Ti
Chemical composition according to the EN 573-1 standard	5.1–6.1	2.1–2.9	1.2–2.0	Max 0.50	Max 0.40	Max 0.30	0.18– 0.28	Zr+Ti Max 0.25	
Results of spectral analysis	5.66	2.2	1.64	0.16	0.22	0.19	0.19	0.06	0.08

**Table 2:** Chemical composition of α-Al_2_O_3_ particles.

Chemical Composition [% Wt]	α-Al_2_O_3_	SiO_2_	Fe_2_O_3_	Na_2_O	CaO	TiO_2_	K_2_O
	>99.0	<0.03	<0.04	<0.19	<0.01	<0.01	<0.01
Density: 3.95 [g/cm^3^]; particle size: 3–6 [μm]							

**Table 3:** Mechanical and physical properties of the manufactured composite materials.

Materials	Density [kg/m^3^]	HBW 2.5/675N	*Rm* [MPa]	*E* [GPa]	*Rg* [MPa]	*Rc* [MPa]	*Rp*_0.2_ [MPa]	Porosity [% vol.]	Impact [kJ/m^2^]
Matrix AW-7075	2810	117	410	81	320	1091	292	1.2	6.7
30% vol.	3150	158	478	142	530	666	396	2.6	2.5
40% vol.	3270	175	448	185	520	563	379	2.9	2.1

where: HBW—hardness Brinell wolfram carbide; *Rm*—tensile strength; *E*—Young’s modulus; *Rg*—bending strength; *Rc*—compressive strength; *Rp*_0.2_—yield strength.

**Table 4:** Chemical composition of 7.62 × 39 FMJ M43 projectile elements.

Element [% Wt]	C	Si	Mn	P	S	Al	Cr	Ni	Cu
Core	0.08	0.04	0.32	0.018	0.027	0.01	0.01	0.01	<0.01
Jacket	0.07	0.03	0.34	0.020	0.024	0.09	0.01	0.01	0.03

**Table 5:** An EDS: Chemical composition at Spectrum 1.

Element	Weight [%]	Atomic [%]
Copper	89.3	89.6
Zinc	10.7	10.4

**Table 6:** Summary of elements used in the simulation.

Element	Type	Size	Quantity
Jacket	Tet	1.0	5038
Core	Tet	1.0	4105
Sample	Tet	1.0	248,125

**Table 7:** Characteristic of material properties of the projectile and materials composites.

Specification	*E* [GPa]	*ν* [-]	*ρ* [kg/m^3^]	*A* [MPa]	*B* [MPa]	*m* [-]	*n* [-]
Core (steel—St45)	210	0.32	7800	430	820	1.03	0.3
Lead jacket (alloy of Pb1 and antimony)	16	0.42	11,270	5.15	3.5	1.03	0.5
Jacket (brass M90)	210	0.33	7800	350	420	1.03	0.3
AW-7075	81	0.3	2810	292	410	1.00	0.4
30% Al_2_O_3_	142	0.25	3150	396	478	1.00	0.3
40% Al_2_O_3_	185	0.23	3270	379	448	1.00	0.27

where: *E*—Young’s modulus; *ν*—Poisson’s ratio; *A*—yield at zero plastic strain; *B*—hardening constant; *m*—temperature softening constant; *n*—hardening exponent.
